# A New Component of the *Nasonia* Sex Determining Cascade Is Maternally Silenced and Regulates *Transformer* Expression

**DOI:** 10.1371/journal.pone.0063618

**Published:** 2013-05-22

**Authors:** Eveline C. Verhulst, Jeremy A. Lynch, Daniel Bopp, Leo W. Beukeboom, Louis van de Zande

**Affiliations:** 1 University of Groningen, Center for Ecological and Evolutionary Studies, Evolutionary Genetics, Groningen, The Netherlands; 2 Universität zu Köln, Institut für Entwicklungsbiologie, Köln, Germany; 3 University of Illinois at Chicago, Departement of Biological Sciences, Chicago, Illinois, United States of America; 4 Institute of Molecular Life Sciences, University of Zurich, Zurich, Switzerland; Centre for Genomic Regulation (CRG), Universitat Pompeu Fabra, Spain

## Abstract

Although sex determination is a universal process in sexually reproducing organisms, sex determination pathways are among the most highly variable genetic systems found in nature. Nevertheless, general principles can be identified among the diversity, like the central role of *transformer* (*tra*) in insects. When a functional TRA protein is produced in early embryogenesis, the female sex determining route is activated, while prevention of TRA production leads to male development. In dipterans, male development is achieved by prevention of female-specific splicing of *tra* mRNA, either mediated by X-chromosome dose or masculinizing factors. In Hymenoptera, which have haplodiploid sex determination, complementary sex determination and maternal imprinting have been identified to regulate timely TRA production. In the parasitoid *Nasonia*, zygotic *transformer* (*Nvtra*) expression and splicing is regulated by a combination of maternal provision of *Nvtra* mRNA and silencing of *Nvtra* expression in unfertilized eggs. It is unclear, however, if this silencing is directly on the *tra* locus or whether it is mediated through maternal silencing of a trans-acting factor. Here we show that in *Nasonia*, female sex determination is dependent on zygotic activation of *Nvtra* expression by an as yet unknown factor. This factor, which we propose to term *womanizer* (*wom*), is maternally silenced during oogenesis to ensure male development in unfertilized eggs. This finding implicates the upstream recruitment of a novel gene in the *Nasonia* sex determining cascade and supports the notion that sex determining cascades can rapidly change by adding new components on top of existing regulators.

## Introduction

Sex determining gene cascades, from the primary signal to the bifunctional switch, are among the most variable developmental systems found in nature [1]. For example, the fruitfly *Drosophila melanogaster* and mammals both have an XX-XY system, but the mechanism that transfers the signal of XX or XY to regulate the onset of sex determination is completely different. In mammals, the Y chromosome carries a dominant male determiner, the SRY gene [2]. In Drosphila, sex determination is X-chromosome dose dependent [3]. Many other Diptera, such as *Ceratitis capitata* and *Musca domestica* also have an XX-XY system, but here the presence of an M-factor blocks the default female mode, thereby promoting male development [4], [5].

The primary signal is processed to regulate sex specific splicing of *transformer* (*tra*) [4], [6]–[8], termed *feminizer* (*fem*) in the honeybee *Apis mellifera* [9]. Female specific *tra* mRNA yields a functional protein, while in males, as a result of early in-frame stop codons, a truncated non-functional TRA protein is produced [4], [7]–[11]. Thus *tra*/*fem* is the central factor in the insect sex determining pathway [12]. The principle of *tra* regulation in diploid insects relies on the paternally inherited genome that prevents female specific splicing in a variety of ways. In a number of dipteran insects a masculinizing (M) factor is transmitted through males only [4], [10], [13]. In *Drosophila* the ratio of X-linked signal elements in XX animals starts the female specific path of the sex-determining cascade by activating *Sexlethal* (*Sxl*), an upstream positive regulator of *tra*. In males, that are XY, the relative level of these signals is insufficient to activate *Sxl* and, hence, *tra* [3], [6]. In Hymenoptera, however, sex is determined by the ploidy of the embryo: males are haploid, developing from unfertilized eggs whereas diploid females develop from fertilized eggs. Until recently, knowledge about primary signals in haplodiploid species was limited to complementary sex determination (CSD) where gender is determined by the allelic state of the *complementary sex determiner* (*csd*) locus or loci [14]. CSD has been inferred for more than 60 hymenopterans [15]. Only in *A. mellifera* however, has the *csd* locus been identified and functionally studied. The *csd* gene (which is a paralog of *fem*) constitutes the primary signal [16]: heterozygosity at the *csd* locus causes female specific splicing of *fem*, which then initiates the female sex determining route. Homozygosity or hemizygosity at the csd locus leads to maleness. As a number of Hymenoptera, including *Nasonia*, do not produce diploid males upon inbreeding [17], another mechanism must lie at the basis of haplodiploid sex determination in these species.

We previously showed an alternative mode of haploidiploid sex determination in *Nasonia* [11]. In this hymenopteran parasitoid, *Nvtra* mRNA is maternally provided to all eggs, similar to what was found in many dipteran insects [4], [10], [13]. However, only in embryos from fertilized eggs is *Nvtra* transcribed in sufficient quantities to initiate and maintain female specific splicing of *Nvtra* by auto regulation, as in all *tra* or fem *fem* containing insects, except *Drosophila* ([12] and refs therein). This supported the hypothesis that the mechanism of sex determination in *Nasonia* is based on maternal silencing of *Nvtra* by genomic imprinting [17], resulting in low expression levels of *Nvtra* in unfertilized eggs that receive only the maternal genome. Fertilized eggs, that receive a paternal genome without the silencing imprint, show high expression levels of *Nvtra*.

Two propositions were presented for the target of imprinting [11]. In a direct scenario, the *Nvtra* gene itself is imprinted during oogenesis to prevent zygotic transcription in haploid offspring. In diploid offspring, resulting from fertilized eggs, only the non-imprinted paternal *Nvtra* allele is transcribed to produce sufficient levels of *Nvtra* transcript to maintain auto regulation. Alternatively, an activator of the *Nvtra* gene could be imprinted on the maternal genome to prevent zygotic *Nvtra* transcription while the non-imprinted paternal activator is needed to activate both *Nvtra* alleles in the zygote to be transcribed and maintain *Nvtra* auto regulation. For diploid early zygotes, the first scenario implies *Nvtra* transcription from the paternal allele only, while in the second scenario *Nvtra* will be transcribed from both the maternal and paternal allele. Which scenario is true can be examined by transcript cloning, using intragenic transcript polymorphisms that distinguish transcripts from the maternal and paternal *Nvtra* allele. One other way to more directly observe whether maternal imprinting at the *Nvtra* locus itself is responsible for the differences in *Nvtra* mRNA levels between unfertilized versus fertilized embryos is to examine the intranuclear patterns of nascent transcription of this gene. It has been shown in *Drosophila* that strong, distinct spots of signal within nuclei detected by *in situ* hybridization correspond to sites of nascent transcription at particular loci [18]. We have adapted a technique for detecting only nascent transcripts in *Nasonia* using anti-sense probes against intronic sequences.

Here, we use both approaches and show that *Nvtra* transcription originates from both the paternal and maternal alleles, indicating that *Nvtra* is regulated by maternal silencing of a trans-acting factor instead of silencing of *Nvtra* itself. This result implicates the upstream recruitment of a novel gene in the *Nasonia* sex determining cascade.

## Results

### Quantitative PCR to Determine Exponential Amplification Stage

**Figure 1:**
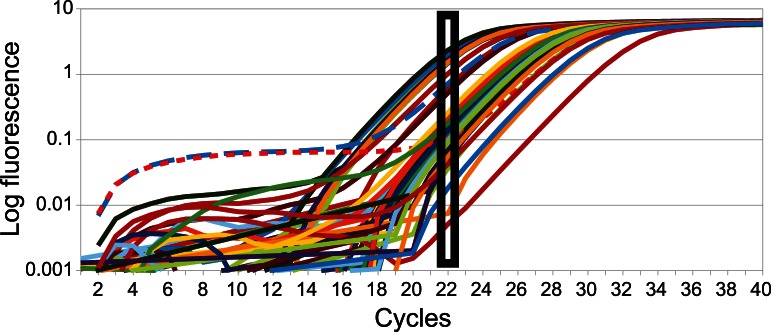
Exponential phase of amplification. Amplification curves of all samples to determine the exact amount of cycles at which all samples would amplify exponentially. The black square indicates the cycles at which all samples are amplifying exponentially. Each line represents one sample.

To reliably determine the relative contribution of the paternal and maternal alleles to zygotic *Nvtra* transcript levels, RT-PCR products must originate from the log-linear phase of the PCR reaction. In this stage any relative difference between maternal and paternal *Nvtra* transcript levels will be reflected in the relative number of PCR products, that subsequently can be cloned and counted. Therefore, a quantitative real-time PCR (qPCR) was performed for each sample. After 22 cycles all samples are within or at the end of their log-linear phase, except for two samples that are starting in this phase (Figure 1). Therefore 22 cycles were used in the PCR step necessary for amplifying the *Nvtra* transcripts for cloning.

### *Nvtra* Expression from the Maternal and Paternal Allele

**Figure 2:**
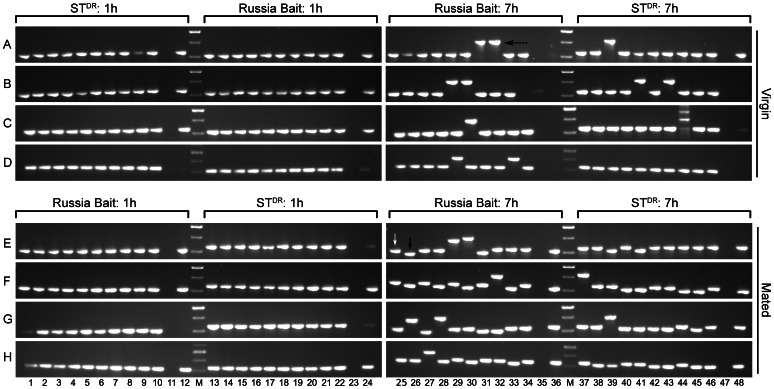
Colony PCR showing the cDNA derived maternal and paternal fragment of *Nvtra*. Panels A – D and E – H indicate the four replicate PCR runs. The maternal origin and age of the embryos is indicated above each set of PCR fragments. White arrow indicates 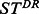 cDNA fragment, black arrow indicates Russia Bait cDNA fragment and dotted arrow indicates gDNA fragment. M is 100 bp molecular standard, ranging from 300 bp (lowest band) to 500 bp (higest band).

The maternal and paternal *Nvtra* transcripts can be distinguished by using a Russia Bait strain that harbours an 18 bp deletion in exon one, 237 bp from the ATG startcodon of *Nvtra* (see also [11]). These size differences can be detected by separation of *Nvtra* mRNA RT-PCR products through a 2% non-denaturing agarose gel. The 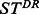 strain is a recessive red eye colour mutant and does not have this deletion. Cloned RT-PCR fragment analysis showed that one hour old embryos produced by both virgin and mated females contain maternally provided *Nvtra* transcripts only, by definition originating from the maternal genome. For both virgin and mated females, one hour old embryos produced by the Russia Bait females have a 328 bp fragment only, while the embryos of the 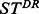 females only have a 310 bp fragment (Figure 2, lane 1–24, all rows). Seven hour old haploid embryos produced by virgin 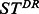 and Russia Bait females show a similar pattern, consistent with the fact that the *Nvtra* fragments can originate only from the maternal allele (Figure 2, row A–D, lane 25–48). In contrast, seven hour old embryos produced by mated 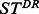 females as well as mated Russia Bait females show *Nvtra* fragments of 328 bp and 310 bp (Figure 2, row E–H, lane 25–48), indicating that the *Nvtra* fragments originate from both the maternal and the paternal allele in these diploid embryos. For the 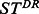 females, 38 fragments were observed, of which 17 were of the Russia Bait (R) allele and 21 from the 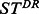 (S) allele (Table 1). For the reciprocal cross with Russia Bait females, in total 34 fragments were observed, containing 14 R alleles and 20 S alleles (Table 1). Results from both reciprocal crosses do not deviate from the expected 50∶50 ratio using a 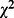-test for goodness of fit (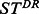 females: p = 0.5164, Russia Bait females: p = 0.3035). In some colony PCR samples a fragment of 416 bp was observed (for example: Figure 2, row A, lane 31–32), indicating a carry over of genomic DNA during RNA extraction.

**Table 1:** Number of maternal and paternal alleles in seven hours old embryos.

	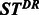 females	Russia Bait females
R allele	17	14
S allele	21	20
Total	38	34

Number of Russia alleles (R) and 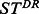 alleles (S) in the seven hours old embryos samples. This number is based on the colony PCR (Figure 2). 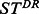 female were mated to Russia Bait males and Russia Bait females were mated to 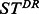 males.

### Nascent *in situ* Hybridisation

One would expect that if one of the *Nvtra* alleles is silenced by imprinting, there would be half as many sites of nascent transcription in comparison with a non-imprinted locus. In addition, one would expect no or very little zygotic transcription from the maternally derived *Nvtra* locus in male embryos. To test these predictions, antisense probes derived from introns of *Nvtra* and the early expressed embryonic patterning gene *Nvcad* [19] were produced, and used to simultaneously detect nascent transcription in early embryogenesis (syncytial blastoderm stages prior to gastrulation), around the time where the difference in *Nvtra* levels were shown to diverge strongly between male and female embryos [11].

We observed strong variation from nucleus to nucleus for both *Nvtra* and *Nvcad*. The number of nuclear spots ranged from 0–4 per nucleus (see methods) for both genes, and the number of *Nvcad* and *Nvtra* spots were often unequal within any given nucleus. The difference in spots for both genes between haploid and diploid embryos is highly significant for both developmental stages (10–11 cycles *Nvtra* 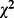 = 41.750, p<0.001; 10–11 cycles *Nvcad* 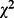 = 51.562, p<0.001; 12 cycles *Nvtra* 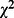 = 75.690, p<0.001; 12 cycles *Nvcad* 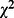 = 139.674, p<0.001). This indicates that both the number of *Nvcad* and *Nvtra* spots is dependent on ploidy level. The difference between *Nvcad* spots in both nuclear division cycles in diploid embryos is significant (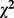 = 21.724, p = 0.04) but the difference in *Nvtra* spots is not (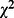 = 10.526, p = 0.570). In haploid embryos the difference of *Nvtra* or *Nvcad* spots is significant in both developmental stages (*Nvtra* 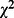 = 14.230, p = 0.03; *Nvcad* 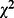 = 31.625, p<0.001). Part of this variability in nuclear spots in both developmental stages is likely derived from differing mitotic states of the nuclei at this very early stage of embryogenesis, where mitoses are taking place at approximately 15–20 minute intervals (J.A. Lynch, in prep). Another explanation may be some instability in upstream inputs into transcription at this early stage of embryogenesis, where maternal control of development is giving way to zygotic control.

**Figure 3:**
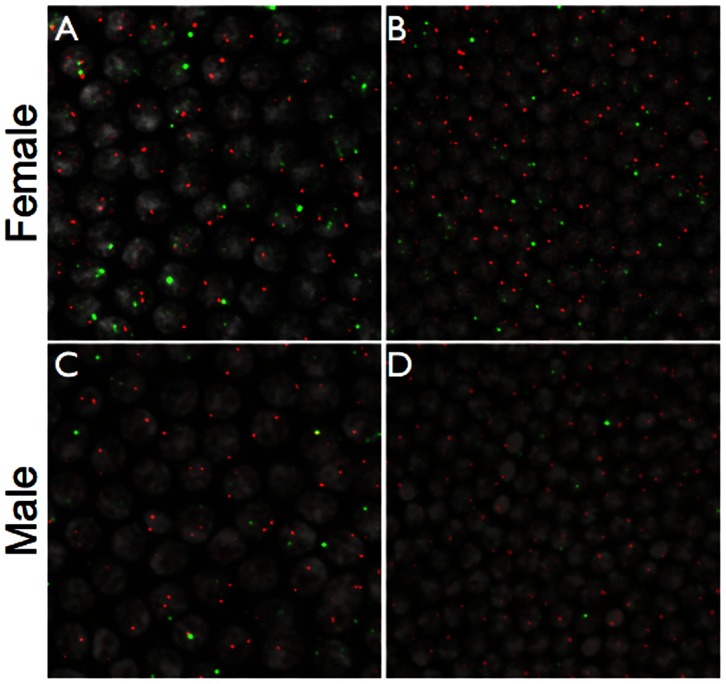
Nascent transcription of *Nvtra* compared to that of *Nvcad*. Nuclear spots of *Nvtra* (green) and *Nvcad* (red) reveal that there is no apparent imprinting of either the *Nvcad* or *Nvtra* locus in early (A) or late (B) blastoderm stage female embryos. In male embryos (C,D) there is moderately less *Nvtra* expression in early blastoderm stage (C), and significantly less in late blastoderm stage (D).

**Figure 4:**
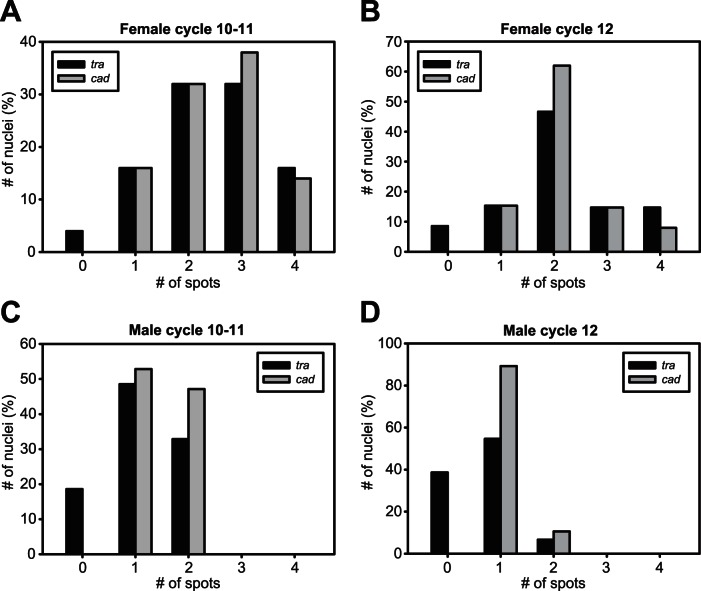
Number of spots of *Nvtra* and *Nvcad* counted in a given number of nuclei (%). Nuclei containing only a *Nvcad* spot are included. Distribution of *Nvtra* (black bars) or *Nvcad* (grey bars) spots in 10–11 cycle female embryos are equal indicating that *Nvtra* and *Nvcad* expression are similar (A). Some variation in the amount of spots is seen which is due to the nuclear division at that stage. In cycle 12 nuclear division is complete (B) and both *Nvtra* and *Nvcad* show primarily diploid expression. In 10–11 cycle male embryos (C) this variation due to nuclear division is also present and after division in cycle 12 *Nvtra* shows haploid expression similar to *Nvcad* (D). In male embryos (C, D) the number of nuclei without a *Nvtra* spot is higher than in female embryos (A, B).

In embryos derived from mated females, of which the eggs should be primarily fertilized and yield diploid females, *Nvtra* nascent transcription is first clearly observed in the early blastoderm stage (approximately division cycles 10 and 11), where it is already strongly expressed (Figure 3A). Despite the observed nucleus to nucleus variability, the average number of sites of transcription for *Nvtra* across multiple nuclei and among multiple embryos were indistinguishable to those of *Nvcad* (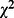 = 0.457, p = 0.928, Figure 4A, Table 2). In later embryos (cycle 12), the intensity of the *Nvtra* spots is reduced, as is the variability among nuclei (Figure 3B), while the average ratio of *Nvtra* to *Nvcad* sites of transcription remained approximately equal (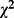 = 4.378, p = 0.223, Figure 4B, Table 2). Since *Nvcad* is expressed from both alleles, this indicates that *Nvtra* is also expressed from both alleles.

**Table 2:** Quantification of nuclear spots.

	# nuclei	# *Nvtra* spots	# *Nvcad* spots	Average *Nvtra* spots/nucleus	Average *Nvcad* spots/nucleus
early female	50	120	125	2.4	2.5
late female	164	346	351	2.11	2.140
early male	75	87	103	1.16	1.373
late male	75	51	83	0.68	1.1

Quantification of nuclear spots of *Nvtra* and *Nvcad* in early and late, male and female embryos. In this table, nuclei containing only a *Nvcad* spot are included.

In embryos derived from virgin females, which will all be haploid males, *Nvtra* expression is also observed. While our methods for *in situ* hybridization are not quantitative, the intensity of *Nvtra* nascent transcription appears qualitatively lower in male embryos in the early stages (Figure 3C, J.A. Lynch personal observation). However, the number of *Nvtra* spots in cycle 10–11 embryos is similar to *Nvcad* spots (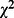 = 0.907, p = 0.341) but Figure 4C and Table 2 show that 18.6% of the nuclei with a *Nvcad* spot do not show a *Nvtra* spot. Embryos that have completed the last syncytial division before gastrulation also have an equal number of *Nvtra* nuclear spots compared to *Nvcad* spots (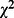 = 0.383, p = 0.536) but again Figure 4D and Table 2 show 38.7% of the nuclei without a *Nvtra* spot. Therefore, these results reflect only rudimentary *Nvtra* transcription during the male syncytial development and are in agreement with earlier results [11].

## Discussion

Using transcript cloning it was shown that in seven hour old diploid embryos *Nvtra* mRNA is transcribed from both parental alleles. By nascent *in situ* hybridization it was shown that in both haploid and diploid embryos the number of *Nvtra* spots is equal to the number of *Nvcad* spots, again indicating that *Nvtra* expression occurs from both alleles in diploid embryos. The intensity of *Nvtra* spots is higher in cycle 10–11 diploid embryos than in cycle 12 diploid embryos which confirms the earlier observation of a peak expression of *Nvtra* in diploid embryos [11]. Moreover, in haploid embryos the expression levels of *Nvtra* seem qualitatively lower when compared to *Nvcad* expression which also corroborates the results of [11]. However, the nascent *in situ* hybridisations also clearly show that appreciable zygotic transcription of *Nvtra* occurs in haploid embryos which may represent basal transcription levels at the *Nvtra* locus. Still, a high number of nuclei in the haploid embryos did not contain a *Nvtra* spot when compared to the number of nuclei in diploid embryos without a *Nvtra* spot (Table 2), which points at patchy expression of *Nvtra* in haploid embryos. This may indicate that *Nvtra* expression in haploid embryos is due to leakage because of an incomplete transcription inhibition at the *Nvtra* gene. Taken together, these results imply that *Nvtra* transcription is regulated by a trans-acting factor, that regulates either the levels of transcription occurring at the *Nvtra* locus, the stability of the resulting mRNA, or a combination of both. This factor is silenced on the maternal chromosome set, and only when fertilized, eggs receive the paternal chromosome with an actively expressed non-silenced factor, that promotes and augments *Nvtra* expression in seven hours old embryos to a level necessary for establishing the autoregulatory feedback loop of *Nvtra* which results in female development.

**Figure 5:**
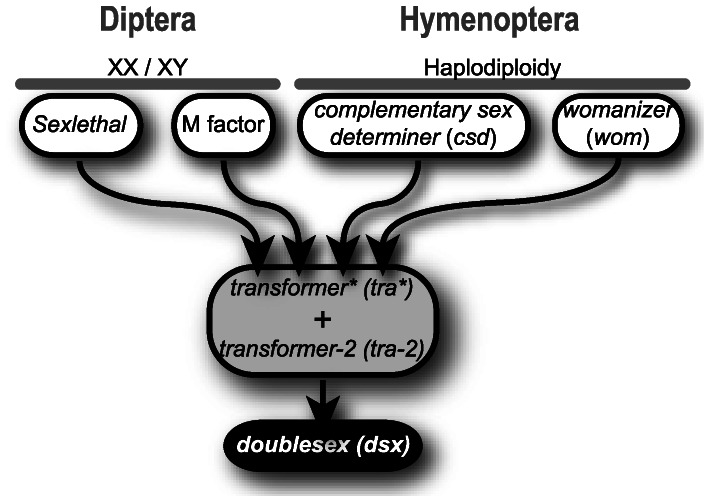
Overview of the different sex determining mechanisms in Diptera and Hymenoptera. The *doublesex* (*dsx*) and *transformer* (*tra*) gene are conserved, but the upstream signal differs between the species (reviewed in [12]). *Drosophila* has *Sexlethal* incorporated upstream of *tra* that splices *tra* and is regulated by the X chromosome dose [3]. Other Diptera (e.g. *C. capitata*, *M. domestica*) have a M-factor usually on the Y-chromosome that blocks *tra* auto regulation [4], [10]. *A. mellifera* has a *complementary sex determiner* (*csd*) that splices *fem* [21] and *Nasonia* has *womanizer* (*wom*) that regulates *tra* expression. In many insect species, like the dipteran *M. domestica*, *Anastrepha obliqua*, *C. capitata*, *Lucilia cuprina*, and *Sciara ocellaris* [13], [35]–[38], the hymenopteran insect Apis mellifera [39] as well as in the lepidopteran insect *Bombyx mori* [40], the *transformer-2* gene is described to be needed for proper *tra* functioning. * indicates that the ortholog of *transformer* is called *feminizer* (*fem*) in *A. mellifera*.

From these results we can infer a novel upstream addition to the sex determination cascade of *Nasonia* that regulates *Nvtra* expression. We propose to term this trans-acting factor *womanizer* (*wom*) (Figure 5). This addition to the *Nasonia* sex determining cascade is in agreement with the theoretical considerations of [20] that sex determining cascades can rapidly change by adding new components to the upstream levels owing to sexual selection. Within the Hymenoptera this evolutionary process is visible when comparing *Nasonia* to *Apis mellifera*. It was shown in *A. mellifera* that the *csd* gene is the result of a duplication of *fem*, and evolved to regulate the sex specific splicing of *fem* in early development [21]. In *Nasonia* the proposed *wom* locus evolved to regulate *Nvtra* expression differentially between fertilized and unfertilized embryos. Between species in the order of Diptera, the regulation of *tra* is also very different, most notably *Drosophila* where *Sex-lethal* was added to the sex determining cascade to regulate *tra* expression [22]. In addition, the M-factors in *Ceratitis capitata* [4] and *Musca domestica* [5] regulate *tra* in an different way by inhibiting *tra* transcription or translation.

The results shown here are in agreement with the maternal effect genomic imprinting sex determination (MEGISD) model as has been suggested by [17] and are an important addition to the findings of [11] on the prominent maternal role in *Nasonia* sex determination. The current results also explain a number of important previous observations on sex determination in *Nasonia*. i) A polyploid mutant strain has been described in which females are triploid and lay haploid and diploid eggs that normally develop into males when unfertilized [23]. The occurence of triploid females that produce diploid males means that we can rule out a ploidy effect in which the number of chromosome sets somehow regulate *Nvtra* expression. ii) [24] generated rare diploid male offspring from X-ray mutagenized wild type haploid males. These biparental diploid males were explained by an imprinting defect in the irradiated paternal germ line generating an epigenetic lesion. In our model, this result is explained by assuming that the X-ray mutagenized males carry an inactive copy of *wom* causing insufficient *Nvtra* transcription in diploid offspring. iii) The paternal-sex-ratio (PSR) chromosome is a supernumerary chromosome in *N. vitripennis* that is transmitted by males only. After fertilization it causes the loss of the paternal genome in the early zygote resulting in male development [25]–[27]. Our model explains why PSR leads to male development: since the paternal genome is condensed quickly after fertilization *wom* cannot act, again resulting in insufficient levels of *Nvtra* expression. iv) The observation of uniparental haploid females and gynandromorphs by [23] can be explained by improper maternal imprinting of *wom* leading to zygotic transcription of *Nvtra* in haploid individuals. Depending on the level of improper imprinting, this would lead to either complete or partial female development of these haploid individuals [28]. We cannot rule out that the proposed *gyn1* locus [29] and the *wom* locus are the same.

The mechanism of maternal silencing of *wom* is as yet unknown. Since DNA methylation has been implicated in gene silencing in insects [30] it is attractive to speculate DNA methylation to be involved in the maternal silencing of *wom*. *Nasonia* possesses a complete gene set of DNA methyltransferases [31] and the importance of maternal provision of DNA methyltransferase *Dnmt1a* in early embryonic development has already been reported [32].

Taken together, our results show that *Nasonia* female sex determination is dependent on zygotic activation of *Nvtra* expression by an as yet unknown factor, that we propose to term *womanizer* (*wom*). This factor, *wom* is maternally silenced upon oogenesis to ensure male development in unfertilized eggs.

## Materials and Methods

### *Nasonia* Strains

The *N. vitripennis* lab strains AsymCx, 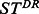, and a Russia Bait strain, originally collected near Moscow (Russia) were used throughout the experiments. All strains have been cured from a Wolbachia infection, cultured under constant light at 25 
C and reared on *Calliphora* sp. fly pupae as hosts.

### Embryo Collection for *Nvtra* Fragment Analysis

Approximately 50 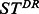 adult females were individually mated to Russia Bait males, and vice versa. They were provided with hosts in egg laying chambers that restricts access of the female to only the anterior part of the fly pupa which greatly facilitates embryo collection. Individual hosting of the mated females also leads to strongly female biased offspring [33]. In addition, virgin 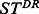 and Russia Bait females were set up individually to produce male offspring only, which is used as control for the presence or absence of the deletion in the Russia Bait or 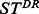 strain, respectively. Females were allowed to feed on one host for 24 hours to stimulate oogenesis, after which they were given a new host for one hour at 25

C that was used for embryo collection. For one hour old embryo samples, embryos were collected immediately and stored in 100% ethanol within 15 minutes. For seven hour old embryo samples, the hosts were incubated for another six hours at 25

C, after which the embryos were collected in a similar way. After collection the embryo samples were stored at 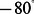
C until RNA extraction. In total six replicates of one hour old embryos and six replicates of seven hour old embryos were collected from mated 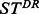 and mated Russia Bait females. Additionally, six replicates of 100 one hour old embryos and six replicates of 100 seven hour old embryos were collected from virgin 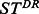 or Russia Bait females.

### RNA Extraction, cDNA Synthesis and Quantitative Real-time PCR

RNA extraction was performed with TriZol according to manufacturers protocol (Invitrogen, Carlsbad, California, USA); during the precipitation step 15 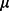
g GlycoBlue™ (Ambion, Austin, Texas, USA) was used to facilitate precipitation of RNA. All isolated total RNA was primed using a mixture of 1∶6 random oligo-dT:random hexamers, both provided with the RevertAid™ H Minus First Strand cDNA Synthesis Kit (Fermentas, Hanover, MD, USA) and reverse transcribed. The resulting cDNA was diluted 1∶100. One 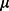
l of this diluted cDNA was used to check the integrity and quality of the cDNA conversion with PCR using 400 nM NvTra_poly_F1 (5′-GGATTGCTTGGATGGTACAG-3′) and NvTra_poly_R1 (5′-TGGATGTTCACTACAACTTGTC-3′) with with 95

C for 3 min, 40 cycles of 95

C for 15 s, 57

C for 30 s and 72

C for 30 s.

For quantitative real-time PCR (qPCR), one 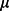
l of 1∶100 diluted cDNA was mixed with 12.5 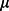
l ABgene ABsolute™ QPCR SYBR® Green ROX (500 nM) Mix (Thermo Fisher Scientific, Germany) and 200 nM of primers NvTra_poly_F1 and NvTra_poly_R1. A standard ABI7300 dissociation curve was applied to check for non-specific amplification.

### RT-PCR Amplification of *Nvtra* Transcript

The primers NvTra_poly_F1 and NvTra_poly_R1 give a fragment of 328 bp in case of *Nvtra* cDNA without the deletion, 310 bp in case of *Nvtra* cDNA with the deletion and 416 bp or 398 bp on genomic DNA [11]. All fragments are confirmed by Sanger sequencing and can be distinguished on a 2% non-denaturing agarose gel containing 0.5
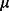
g/ml ethidium bromide.

From the eight sample classes each replicate was used four times in a PCR reaction with similar parameters as described for the qPCR. In this way any bias of PCR amplification is averaged over four PCR runs. One 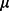
l 1∶100 diluted cDNA was mixed with 10x PCR buffer (Roche) (10x concentrate; 100 mM Tris-HCl, 500 mM KCl; pH 8.3), 200 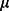
M dNTPs, 2 units Taq polymerase (Roche), 400 nM of primers NvTra_poly_F1 and NvTra_poly_R1 and supplemented with milliQ water to 25 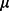
l. PCR profile was as follows: initial denaturing step of 95

C for 3 min, followed by 20 cycles of 95

C for 15 s, 57

C for 30 s and 72

C for 30 s, ending with a final extension step of 72

C for 7 min. As negative control one 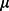
l of milliQ water was used. Since the amount of product was too low to visualize on gel, all six replicates for each sample class were pooled and one 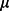
l of this was used in a similar PCR regime as above but with 40 cycles. In this way the samples could be checked for improper amplification and possible contaminations on a 2% non-denaturing agarose gel with 0.5
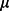
g/ml ethidiumbromide. The negative control samples from the 22 cycles PCR were also used in this PCR as templates to check for reagent and water contaminations.

### Cloning PCR Fragments

For each PCR reaction, the six replicate samples were pooled, so that each sample class consisted of four samples, that were subsequently purified using the GeneJET PCR Purification Kit (Fermentas, Hanover, MD, USA) to remove primers, nucleotides and salt. After PCR clean up, samples were checked again for improper amplification using a 40 cycles PCR as described above. Using a nanodrop2000 (Nanodrop, Wilmington, DE, USA) the purity and concentration of each sample was determined. The four samples, per sample class, were pooled, resulting in one sample for each of the eight sample classes. Approximately 10 ng of each sample was ligated into a pGEM®
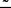
T vector (Promega, Madison, WI, USA) according to manufacturers protocol. JM-109 *E. coli* (efficiency: 1×10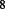 cfu/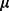
g DNA) cells that were supplied with the pGEM®
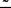
T Vector System II (Promega, Madison, WI, USA) were transformed with five 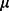
l of ligation reaction, and plated onto 2xYT, 100 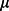
g/ml ampicillin, 80
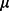
g/ml X-gal, 0.5 mM IPTG, agar plate. These plates were incubated for 16 hours at 37

C and stored at 4

C for 2 hours to facilitate blue/white screening. Forty white colonies and four blue colonies from each sample point were picked up and used in a colony PCR as described in the section above. The resulting PCR fragments were visualized on a 2% agarosegel with 1x TBE buffer with 0.5
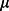
g/ml ethidiumbromide. 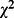 tests of goodness of fit were performed in Microsoft Excel.

### Nascent Transcript *in situ* Hybridisation

For *in situ* hybridisations, the reference gene *N. vitripennis caudal* (*Nvcad*) was chosen as comparison for *Nvtra* expression, because it has a strong, and initially broad early zygotic domain of expression [19].

Fragments of *Nvcad* and *Nvtra* introns were isolated using the following primers:

- nvtraintF1 ggccgcggTACGCTCAGGTGCTAACTGC
- nvtraintR1 cccggggcTTTATGCATGAATGGCCAAC
- nvcadintF ggccgcggCAAGCCGTTCTCCCATTAAA
- nvcadintR cccggggcACAGCGAAAAACGAGAGGAA

These fragments were then used as the basis for *in situ* hybridization probe synthesis. AsymCx wasps were allowed to lay eggs in a Waspinator chamber (J.A. Lynch, in prep) for 3 hours at 29 
C. Eggs were then aged for two hours and immediately fixed, and hand dechorionated by the method of [34]. The developmental stages present in this population of embryos overlaps those used for the PCR based analysis of *Nvtra* expression. *In situ* hybridizations were performed with a standard protocol with some minor modifications (detailed protocol can be obtained on request from the authors (J.A. Lynch)). Digoxigenin (dig) labeled probes were detected using an anti-dig::POD antibody (1∶100) followed by fluorescent amplification using the AlexaFluor 488 tyramide kit from Life technologies. Dinitrophenyl (DNP) labeled probes were detected with rabbit anti-DNP antibody (1∶300) followed by an anti-rabbit Alexa 568 labelled secondary antibody (1∶500). Embryos were mounted in Vectashield with DAPI. Images shown were produced using a dig labelled *Nvtra* intron probe in combination with a DNP *N. vitripennis caudal* (*Nvcad*) probe. Experiments where the labels were exchanged gave similar results.

### Counting Nuclear Dots

Confocal images of stained embryos were obtained using a Zeiss LSM 710 microscope using the Zen software package. Sections were flattened into projections using the Standard Deviation projection function in Image J. Projections of each channel were overlaid and adjusted for contrast in Adobe Photoshop. Twenty-five to thirty-five nuclei from each embryo were analyzed. Nuclei that had more than the expected maximum number (4, for diploid embryos where S-phase of mitosis has been completed) of spots for either gene were excluded from analysis. Such nuclei were infrequent, and likely arose due to technical artifacts, or due to the presence of large aggregates of mRNA that do not represent nascent transcription, but rather some other regulatory process acting on completely transcribed mRNAs (such as regulated export from the nucleus, or localized areas of transcript processing). Only spots that completely overlapped with a nucleus were counted. Finally, only nuclei that showed at least one dot of *Nvcad* expression were analyzed. Analysed nuclei were generally located toward the posterior pole, as this is the main domain of expression of *Nvcad* (J.A. Lynch, personal observation). 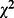 tests of independence were performed in Microsoft Excel. To prevent bias due to the removal of non- *Nvcad* expressing nuclei, P values were calculated only on the nuclei that showed both *Nvtra* and *Nvcad* spots.
